# A Nomogram for Predicting the Recurrence of Acute Non-Cardioembolic Ischemic Stroke: A Retrospective Hospital-Based Cohort Analysis

**DOI:** 10.3390/brainsci13071051

**Published:** 2023-07-10

**Authors:** Kangmei Shao, Fan Zhang, Yongnan Li, Hongbin Cai, Ewetse Paul Maswikiti, Mingming Li, Xueyang Shen, Longde Wang, Zhaoming Ge

**Affiliations:** 1 Department of Neurology, Lanzhou University Second Hospital, Lanzhou 730030, China; shaokm21@lzu.edu.cn (K.S.); caihb407@163.com (H.C.); limm20@lzu.edu.cn (M.L.); 120220901820@lzu.edu.cn (X.S.); 2 Gansu Provincial Neurology Clinical Medical Research Center, Lanzhou University Second Hospital, Lanzhou 730030, China; 3 Department of Oncology Surgery, Lanzhou University Second Hospital, Lanzhou 730030, China; zhangf16@lzu.edu.cn (F.Z.); ewetse2019@lzu.edu.cn (E.P.M.); 4 Department of Cardiac Surgery, Lanzhou University Second Hospital, Lanzhou 730030, China; lyngyq2006@foxmail.com; 5 Expert Workstation of Academician Wang Longde, Lanzhou University Second Hospital, Lanzhou 730030, China; longde_wang@yeah.net

**Keywords:** non-cardiogenic ischemic stroke, recurrence, risk factors, nomogram, prediction model

## Abstract

Non-cardioembolic ischemic stroke (IS) is the predominant subtype of IS. This study aimed to construct a nomogram for recurrence risks in patients with non-cardioembolic IS in order to maximize clinical benefits. From April 2015 to December 2019, data from consecutive patients who were diagnosed with non-cardioembolic IS were collected from Lanzhou University Second Hospital. The least absolute shrinkage and selection operator (LASSO) regression analysis was used to optimize variable selection. Multivariable Cox regression analyses were used to identify the independent risk factors. A nomogram model was constructed using the “rms” package in R software via multifactor Cox regression. The accuracy of the model was evaluated using the receiver operating characteristic (ROC), calibration curve, and decision curve analyses (DCA). A total of 729 non-cardioembolic IS patients were enrolled, including 498 (68.3%) male patients and 231 (31.7%) female patients. Among them, there were 137 patients (18.8%) with recurrence. The patients were randomly divided into training and testing sets. The Kaplan–Meier survival analysis of the training and testing sets consistently revealed that the recurrence rates in the high-risk group were significantly higher than those in the low-risk group (*p* < 0.01). Moreover, the receiver operating characteristic curve analysis of the risk score demonstrated that the area under the curve was 0.778 and 0.760 in the training and testing sets, respectively. The nomogram comprised independent risk factors, including age, diabetes, platelet–lymphocyte ratio, leukoencephalopathy, neutrophil, monocytes, total protein, platelet, albumin, indirect bilirubin, and high-density lipoprotein. The C-index of the nomogram was 0.752 (95% CI: 0.705~0.799) in the training set and 0.749 (95% CI: 0.663~0.835) in the testing set. The nomogram model can be used as an effective tool for carrying out individualized recurrence predictions for non-cardioembolic IS.

## 1. Introduction

Ischemic stroke (IS) is one of the most common and prevalent stroke subtypes, accounting for more than 80% of patients diagnosed with stroke [1]. Data from the Global Burden of Disease study demonstrated that 77.19 million people had IS in 2019, including 7.63 million new-onset IS cases, and an additional 3.29 million mortalities worldwide [2]. To date, globally, a meta-analysis estimated that the 1-year and 5-year recurrence rates of IS are 10.4% and 14.8%, respectively [3]. Non-cardioembolic IS, which accounts for more than 70% of IS, is primarily caused by large arterial atherosclerosis or small-vessel occlusion [4]. The Chinese population is more likely to have non-cardioembolic IS, such as lacunar stroke, which results from small-vessel diseases [5]. Recurrent patients exhibit more severe symptoms and higher disability risks and mortality rates [6]. Several studies have reported that the 1-year recurrence rate of non-cardioembolic IS is 7.1–11.0% [7,8]. Therefore, predictions of the recurrence risk for non-cardioembolic IS patients at early stages could reduce recurrence rates, death risks, disability probabilities and the resultant increased utilization of healthcare resources.

However, as of recently, only a small amount of information is available in the literature concerning the prediction models for evaluating the recurrence risk of non-cardioembolic IS patients. The Essen Stroke Risk Score (ESRS) model is a 10-point scale derived and validated to evaluate the recurrence risk of IS patients who had non-atrial fibrillation [9]. To verify this prediction efficacy, several clinical investigations reported that the C-index of the ESRS model was 0.55–0.60 [10,11,12,13]. Moreover, the Stroke Prognosis Instrument (SPI)-I model was proposed mainly to predict the risk of recurrence or death within 2 years post-IS disease processes, but not for the non-cardioembolic IS subtype [14]. The predictive efficacy of SPI-II, which is a modified version of SPI-I, was better than that of SPI-I, but the efficacy was still not high (the area under the curve (AUC) was 0.63), especially for short-term recurrence risk [15]. The ABCD/ABCD2 and ABCD 3-I models were used to evaluate (transient ischemic attack, TIA) patients with a high risk for IS [16,17,18]. The recurrence risk estimator at 90 days (RRE-90) model performed well in predicting the 90-day recurrent stroke risk of IS [19]. As a result, there is a great need to construct a novel model for the long-term recurrence risk prediction of non-cardioembolic IS patients.

In this study, we attempt to identify the independent risk factors and construct a nomogram to accurately evaluate the long-term recurrence risk for non-cardioembolic IS patients by retrospectively enrolling non-cardioembolic IS patients in our health center. This study may contribute to the efficient selection of non-cardioembolic IS patients with high recurrence risk and provide optimal secondary prevention treatment strategies for these patients.

## 2. Materials and Methods

### 2.1. Patients Enrollment and Study Design

**Figure 1:**
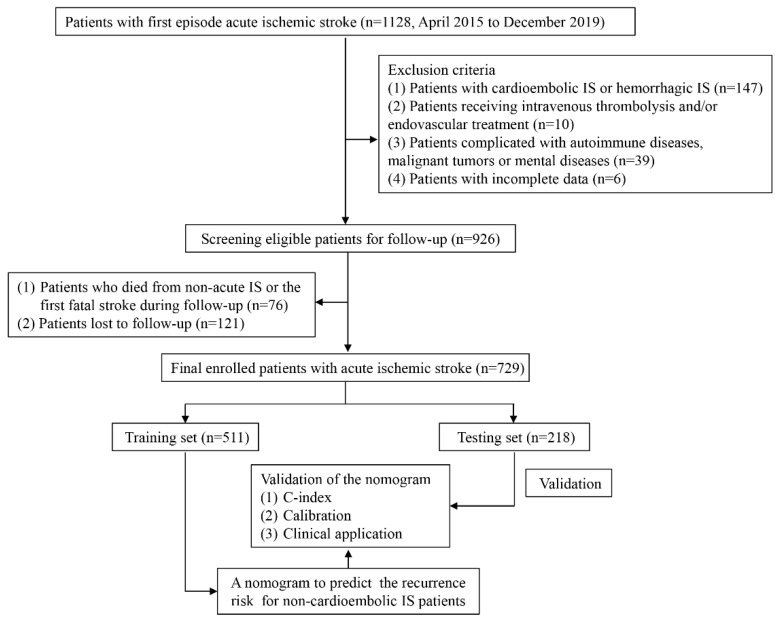
Flow chart of the selection of eligible patients.

This retrospective study was conducted at the stroke center of Lanzhou University Second Hospital from April 2015 to December 2019. The inclusion criteria were as follows: (1) age ≥ 18 years; (2) first-episode IS patients with new lesions and no old lesions confirmed by cranial computed tomography (CT) scan and/or cranial magnetic resonance imaging (MRI); (3) patients admitted within 2 weeks of symptom onset; (4) all patients were treated conservatively. The exclusion criteria were as follows: (1) patients with cardioembolic stroke or hemorrhagic stroke, the diagnosis of which being made according to the established diagnostic criteria [20,21]; (2) patients receiving intravenous thrombolysis and/or endovascular treatment, which are time-sensitive interventions, and their clinical presentations and prognoses are distinct from medical management [22,23]; (3) patients complicated with autoimmune diseases, malignant tumors or mental diseases; (4) patients with incomplete data. A total of 926 patients with non-cardioembolic IS were consecutively enrolled, and they followed up until November 2020. The primary end point was the recurrence of non-cardioembolic IS. In addition, recurrence was identified using CT/MRI scans and examining clinical manifestations. After follow-up, 76 patients died from non-acute IS or the first fatal stroke, and 121 patients lost to follow-up were eventually excluded from the present study. The details of the inclusion and exclusion criteria are shown in Figure 1. Furthermore, the study has been approved by the Ethics Committee of Lanzhou University Second Hospital.

### 2.2. Data Collection

Clinical information and laboratory data were obtained from the hospital information system (HIS) of Lanzhou University Second Hospital, including demographic information, previous history, personal history, laboratory examination results, imaging examinations, and inflammatory factors. The classification of drug compliance was in accordance with the follow-up outcomes. Diabetes was defined as self-reported, physician-diagnosed diabetes and/or the use of insulin or oral hypoglycemic medications, a fasting serum glucose ≥ 126 mg/dL, or a non-fasting serum glucose ≥ 200 mg/dL. Hypertension was defined as self-reported, physician-diagnosed hypertension in addition to the use of antihypertensive medication, systolic blood pressure ≥ 140 mmHg, or diastolic blood pressure ≥ 90 mmHg [24]. Chronic kidney disease (CKD) was defined as eGFR < 60 mL/min per 1·73 m^2^ [25]. Patients with carotid atherosclerosis (CAS) were defined according to the Mannheim consensus [26]. CAS could be categorized into the following groups according to the results of cervical vascular ultrasound examinations: none, carotid intima-media thickness (CIMT), stable plaques, unstable plaques, and carotid stenosis. Patients with leukoencephalopathy could be classified into none, Fazekas 1 grade, Fazekas 2 grade and Fazekas 3 grade according to the severity of white-matter hyperintensities in MRI using the Fazekas scale [27]. According to body mass index (BMI), patients were classified into BMI < 18, 18 ≤ BMI < 24, 24 ≤ BMI < 28, and BMI ≥ 28. Hyperlipidemia was defined as total triglycerides (TGs) > 1.7 mmol/L and/or total cholesterol (TC) > 5.17 mmol/L and/or low-density lipoprotein cholesterol (LDL-C) ≥ 3.37 mmol/L, or a history of hyperlipidemia [28]. Patients with hypertension were divided into none, adequately controlled hypertension, and inadequately controlled hypertension. Patients with diabetes were differentiated as none, adequately controlled diabetes, and inadequately controlled diabetes.

According to the follow-up data, patients were categorized as not receiving aspirin therapy and those receiving aspirin therapy. In addition, patients were categorized as not receiving statin medication therapy and receiving statin medication therapy. The neutrophil-to-lymphocyte ratio (NLR), platelet-to-lymphocyte ratio (PLR), lymphocyte-to-monocytes ratio (LMR), and systemic immune inflammation index (SII) were inflammatory markers. The SII was defined as platelet × neutrophil/lymphocyte.

### 2.3. Primary Outcome Assessment

The primary end point was the recurrence of non-cardioembolic IS. To obtain primary end-point data, a telephone or face-to-face follow-up was conducted after obtaining consent from patients or their relatives.

### 2.4. Screening Recurrence Risk Factors for Non-Cardioembolic IS Patients

To avoid selection bias, all patients were randomly divided into training and testing sets according to a 7:3 ratio using the “caret” package in R software. In the training set, we utilized the univariate Cox regression analysis to screen potential risk factors of the recurrence of non-cardioembolic IS. Then, we performed the least absolute choice operator (LASSO) Cox regression with ten-fold cross-validation and a *p*-value of 0.05 using the “glmnet” package in R based on the variables obtained from the univariate analysis. Subsequently, the variables screened by the LASSO Cox regression were incorporated into the multivariate Cox regression model, and the variables with a *p*-value under 0.05 were used to construct a recurrence risk model [29]. The hazard ratio (HR) and the 95% confidence interval (CI) of the independent risk factors were represented using a forest map in R. The recurrence risk scores were calculated based on the variables weighted by the regression coefficient in multivariate Cox regression analysis. The recurrence risk scores were calculated as follows: recurrence risk scores = 0.478 × age (<65, 0; ≥65, 1) + 0.235 × leukoencephalopathy (no, 0; Fazekas grade 1, 1; Fazekas grade 2, 2; Fazekas grade 3, 3) + 0.378 × diabetes (no, 0; adequately controlled, 1; inadequately controlled, 2) + 1.134 × PLR (<66.17, 0; ≥66.17, 1) + 0.693 × NE (<5.55 × 10^9^/L, 0; ≥5.55 × 10^9^/L, 1) − 0.621 × MO (<0.35 × 10^9^/L, 0; ≥0.35 × 10^9^/L, 1) − 0.539 × PLT (<185 × 10^9^/L, 0; ≥185 × 10^9^/L, 1) + 0.691 × TP (<71.8 g/L, 0; ≥71.8 g/L, 1) − 0.632 × ALB (<41.30 g/L, 0; ≥41.30 g/L, 1) + 0.668 × IBIL (<16.9µmol/L, 0; ≥16.9µmol/L, 1) − 0.744 × HDL (<1.11 mmol/L, 0; ≥1.11 mmol/L, 1). We divided non-cardioembolic IS patients into high- and low-risk groups based on the median risk scores in the training set. We analyzed the difference in the recurrence of non-cardioembolic IS patients’ high and low risk scores using the “survminer” package in R software [30]. To evaluate the accuracy of the risk model, we constructed ROC curves using the “survival ROC” package in both training and testing sets, respectively [31].

### 2.5. Construction and Validation of Nomogram

According to the independent risk factors, a nomogram was constructed using the “rms” package in R software to predict the 3-, 4-, and 5-year non-recurrence risk of non-cardioembolic IS patients. The nomogram was subjected to the 1000-times bootstrap method for internal validation and external validation. The consistency index (C-index) was utilized to evaluate the prediction ability of the nomogram. The closer the C-index is to 1, the stronger the prediction ability of the nomogram. A calibration plot was used to verify the consistency of the nomogram, and decision curve analysis (DCA) was used to determine the clinical application value of the nomogram. The ROC curve of AIS recurrence was obtained to evaluate the predictive sensitivity and accuracy of the nomogram confirmed in training and testing sets.

### 2.6. Statistical Analysis

The statistical review of the present study was performed by a biomedical statistician. Statistical analysis was performed using R software (version 4.0.3; Boston, MA, USA) and SPSS 24.0 software. The study population characteristics were presented as the mean ± standard deviation (SD), or percentage. For continuous variables, X-tile software was used to determine the best cutoff value and was transformed into categorical variables. Differences in the categorical variables were compared by Chi-square tests. All the test results adopted a two-tailed test, and a *p*-value < 0.05 was considered statistically significant. The risk factors (*p*-value < 0.05) obtained by the multivariate Cox regression analysis were incorporated into the nomogram models. The nomogram models were constructed using a training cohort (70%) to predict recurrence risk. A validation cohort (30%) was used to evaluate the generalization ability of models and was quantitatively evaluated using the AUC and C-index. The values of the C-index and AUC > 0.7 suggest a reasonable estimation.

## 3. Results

### 3.1. Patient Characteristics

A total of 729 non-cardioembolic IS patients were finally included in this study, including 498 (68.3%) male patients and 231 (31.7%) female patients. Among them, 373 (51.2%) patients were younger than 65 years old, and 356 (48.8%) patients were 65 years or older. After follow-up, there were 137 (18.8%) patients with recurrence. The details of the clinical characteristics of non-cardioembolic IS patients are shown in Table 1. Therefore, the cohort was randomly divided into training and testing sets. In addition, the training set included 511 patients, and the testing set included 218 patients. No significant differences with respect to clinical data were observed between the training and testing sets, except for hypertension (*p* < 0.05), as presented in Table 1.

### 3.2. Identification of Risk Factors for Recurrence in Non-Cardioembolic IS Patients

**Figure 2:**
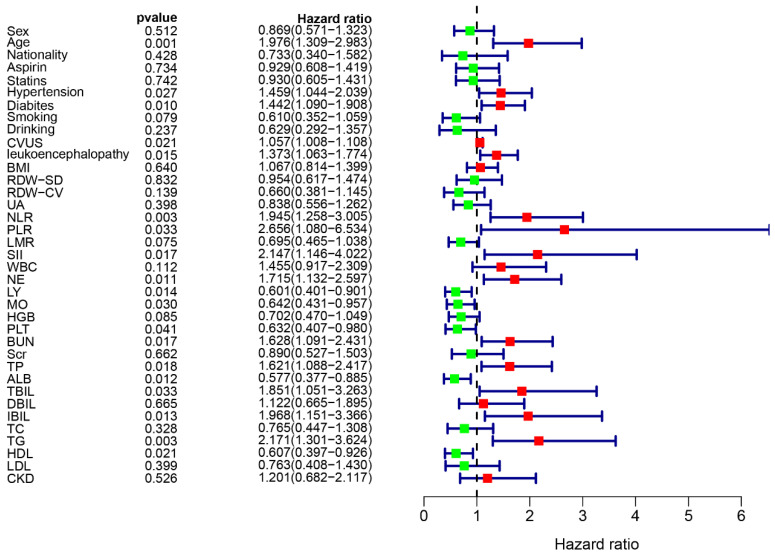
Forest map of the univariate Cox hazard model for risk factors.

**Figure 3:**
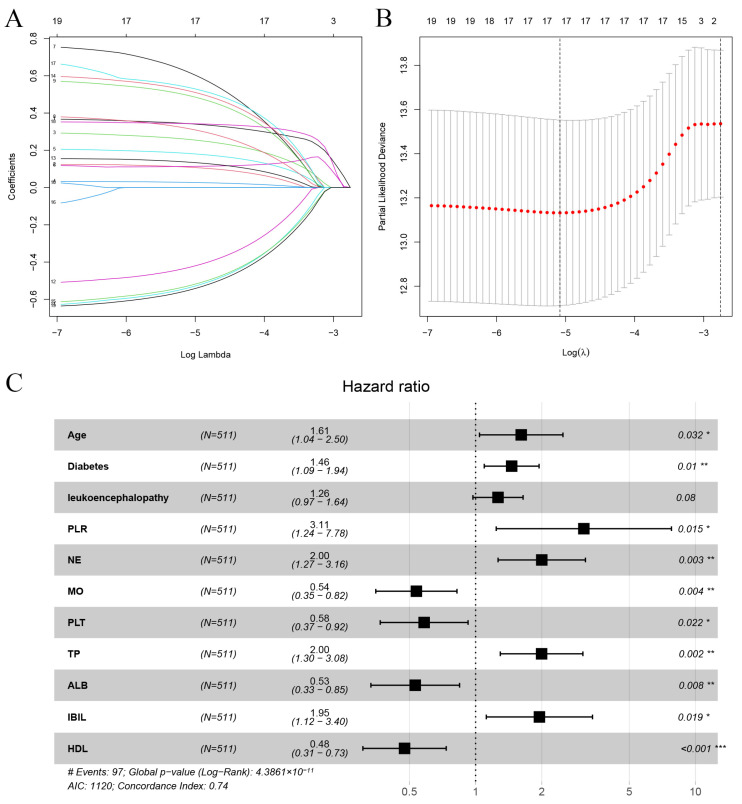
Identification of main contributors to recurrence. (**A**) Optimum parameter (lambda) selection in the LASSO model by ten-fold cross-validation. Color lines: the risk factors from univariate Cox regression analysis. (**B**) Profiles of the LASSO coefficients for the 19 candidate variables. Red dots: different values of λ corresponding to the residual valuables in equation. (**C**) Forest map of the multivariate Cox hazard model for risk factors. Black square: HR for each variable; black horizontal line: 95% CI for HR; *: *p* < 0.05; **: *p* < 0.01; ***: *p* < 0.001.

To screen the risk factors related to the recurrence of non-cardioembolic IS, we attempted to enroll more clinical parameters in our cohort. Following that, the univariate Cox regression analysis was used to demonstrate that the non-cardioembolic IS recurrence was associated with 19 risk factors, as illustrated in Figure 2. This included age, TG, diabetes, neutrophil (NE), NLR, SII, PLR, leukoencephalopathy, albumin (ALB), indirect bilirubin (IBIL), total bilirubin (TBIL), lymphocyte (LY), blood urea nitrogen (BUN), total protein (TP), high-density lipoprotein (HDL), CAS, hypertension, monocytes (MOs), and platelet (PLT). Furthermore, LASSO regression analysis was used to identify variables with the most predictive values for recurrence. Subsequently, 17 variables with non-zero coefficients were screened from the above 19 variables. The variables comprised age, hypertension, diabetes, CAS, leukoencephalopathy, NLR, PLR, SII, NE, MO, PLT, BUN, TP, ALB, IBIL, TG, and HDL. There was no severe collinearity existing in these variables, indicating that these could be utilized to construct a prediction model (Figure 3A,B).

To identify the independent risk factors, the multivariable Cox regression analysis showed that age (HR = 1.61, 95% CI = 1.04~2.50, *p* = 0.032), diabetes (HR = 1.46, 95% CI = 1.09~1.94, *p* = 0.010), PLR (HR = 3.11, 95% CI = 1.24~7.78, *p* = 0.015), leukoencephalopathy (HR = 1.26, 95% CI = 0.97~1.64, *p* = 0.080), NE (HR = 2.00, 95% CI = 1.27~3.16, *p* = 0.003), MO (HR = 0.54, 95% CI = 0.35~0.82, *p* = 0.004), PLT (HR = 0.58, 95% CI = 0.37~0.92, *p* = 0.021), TP (HR = 2.00, 95% CI = 1.30~3.08, *p* = 0.002), ALB (HR = 0.53, 95% CI = 0.33~0.85, *p* = 0.007), IBIL (HR = 1.95, 95% CI = 1.12~3.40, *p* = 0.019) and HDL (HR = 0.47, 95% CI = 0.31~0.73, *p* = 0.001) were significantly associated with recurrence in non-cardioembolic IS patients. Additionally, the forest map was incorporated to visualize the *p*-value, HR and 95% CI of each recurrence-related risk factor (Figure 3C).

### 3.3. Risk Stratification and Recurrence Assessment

**Figure 4:**
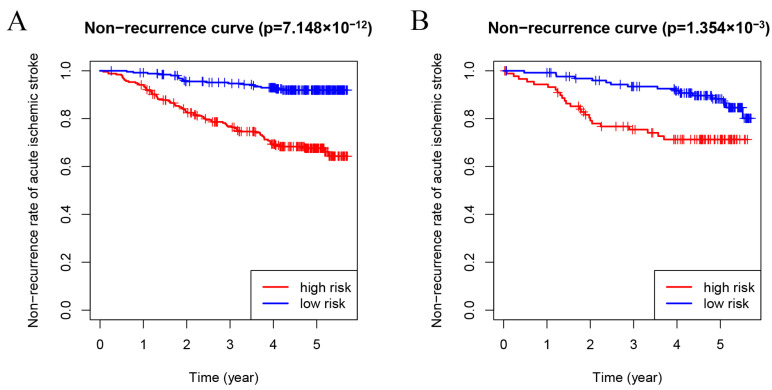
Risk stratification analysis of the recurrence model for non-cardioembolic IS patients. (**A**) and (**B**) Kaplan–Meier analyses showing that non-cardioembolic IS patients with high-risk scores had a higher recurrence rate than those with low-risk scores in the training and testing sets.

Subsequently, in order to further evaluate the difference in recurrence between low-risk and high-risk groups in the training set, a Kaplan–Meier survival analysis was used, and it was observed that the recurrence rate in the high-risk group was significantly higher than that in the low-risk group (*p* = 7.148 × 10^−12^, Figure 4A).

### 3.4. Validation of the Recurrence Prediction Model for Non-Cardioembolic IS Patients

To verify the predictive value of the risk scoring model, the recurrence rate in the testing set was consistent with those in the training set as shown by the Kaplan–Meier survival analysis (*p* = 1.354 × 10^−3^, Figure 4B).

### 3.5. Construction and Validation of the Nomogram to Predict the Recurrence Probability for Non-Cardioembolic IS

**Figure 5:**
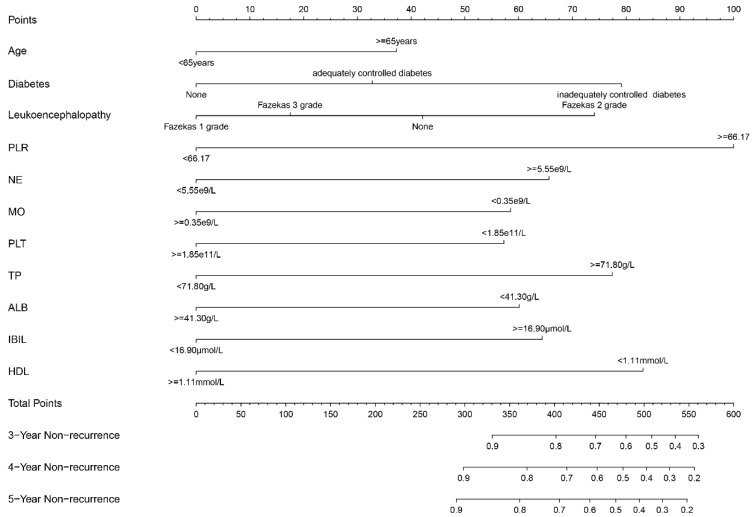
Nomogram for predicting the risk of non-recurrence in patients with non-cardioembolic IS.

Based on the multivariate Cox regression analysis, the nomogram comprised 11 parameters, including age, diabetes, PLR, leukoencephalopathy, NE, MO, TP, PLT, ALB, IBIL, and HDL. The nomogram could predict the non-recurrence risk at 3, 4, and 5 years, as shown in Figure 5. The total points were calculated by summing the score of each prediction variable, and the predicted risk corresponding to the total score was the probability of non-recurrence risk.

**Figure 6:**
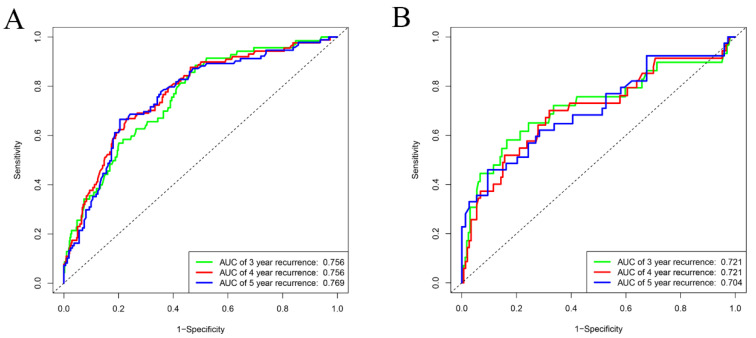
ROC curve of the nomogram in the training set (**A**) and testing set (**B**). The yellow line represents 3-year non-recurrence rate. The green line represents 4-year non-recurrence rate. The blue line represents 5-year non-recurrence rate.

**Figure 7:**
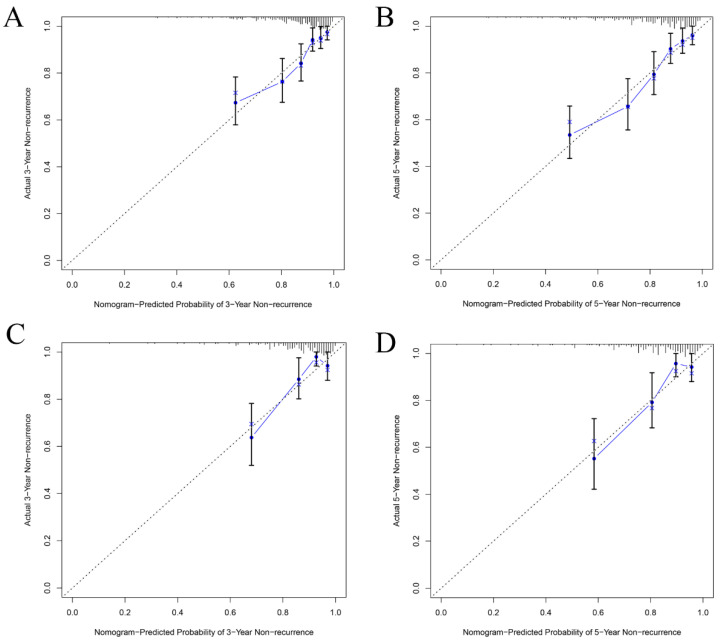
Calibration curves for the 3-year and 5-year non-recurrence of non-cardioembolic IS patients in the training (**A**,**B**) and testing sets (**C**,**D**).

The C-index of the nomogram was 0.752 (95% CI: 0.705~0.799) in the training set for the non-recurrence rate, demonstrating that the nomogram had a higher prediction accuracy. To further validate the prediction efficacy of the nomogram, the C-index of the nomogram in the testing set was 0.749 (95% CI: 0.663~0.835). In addition, the ROC curve showed that the AUC for predicting the recurrence in 3-year, 4-year, and 5-year cases were 0.756, 0.756, and 0.769 in the training set, respectively (Figure 6A). The AUC of the testing set for predicting non-recurrence in 3-year, 4-year, and 5-year cases were 0.721, 0.721, and 0.704, respectively (Figure 6B). Both the AUC and C-index value indicated that the new predictive model is suitable for accurately predicting the risk of non-recurrence. The 3- and 5-year calibration curves showed the best consistency between the predicted and actual non-recurrence risk in the training (Figure 7A,B) and testing sets (Figure 7C,D). The nomogram also indicated adequate prediction accuracy.

**Figure 8:**
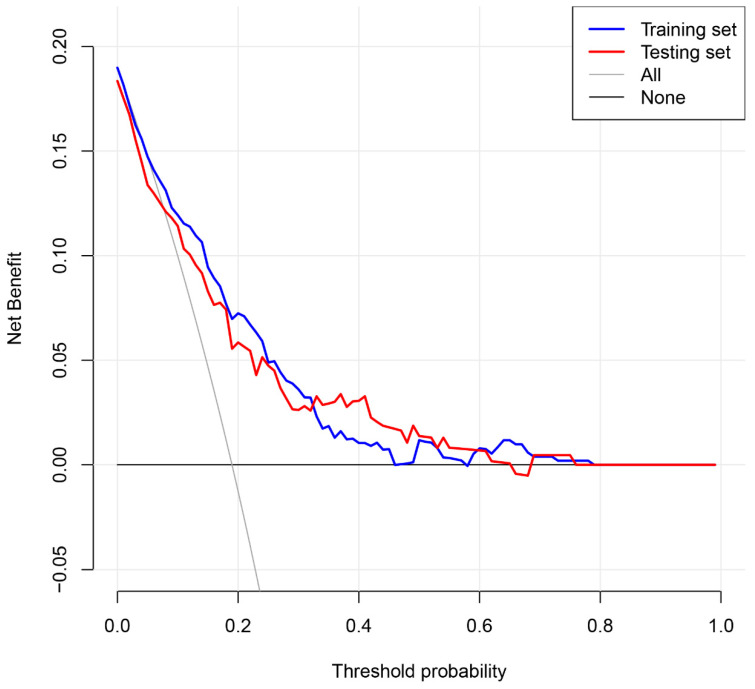
Decision curve analysis (DCA) for recurrence of non-cardioembolic IS patients. The vertical axis measures the standardized net benefit. The horizontal axis shows the corresponding threshold probability. DCA showed that predicting recurrence for non-cardioembolic IS by the nomogram model would be better than having all patients or no patients treated by this model with a range of threshold probability between >0.01 and <0.59 in the training set and between >0.01 and <0.65 in the testing set.

The aim was to identify the clinical applications of the nomogram via quantifying the net benefits at different threshold probabilities. Decision curve analysis (DCA) is a method to evaluate prognostic strategies. DCA showed that predicting recurrence for non-cardioembolic IS by the nomogram model would be better than having all patients or no patients treated by this model with a range of threshold probability between >0.01 and <0.59 in the training set and between >0.01 and <0.65 in the testing set. The results demonstrated that the nomogram model showed a good clinical application value over a wide range of risks of recurrence in both the training and testing sets (Figure 8).

## 4. Discussion

Our study enrolled 729 non-cardioembolic IS patients in total to construct a nomogram that could accurately evaluate the long-term non-recurrence risk. Moreover, in our study, the nomogram was built using predictive variables, including age, diabetes, PLR, leukoencephalopathy, NE, MO, PLT, TP, ALB, IBIL and HDL. This model displayed an internally validated C-index of 0.752 (95% CI: 0.705~0.799) and an externally validated C-index of 0.749 (95% CI: 0.663~0.835), which is higher than that of the ESRS (C-index = 0.613, 95% CI: 0.565–0.661) and SPI-II (C-index = 0.613, 95% CI: 0.564–0.662) models for the prediction of 5 year-recurrence risk [32]. This study demonstrated that our model could be utilized to predict the long-term non-recurrence risk for non-cardioembolic IS patients more accurately and effectively.

Among all the independent risk factors, age was reported as the most important non-modifiable risk factor for IS [33]. However, using age as a risk factor for IS recurrence has been controversial. Numerous research studies have confirmed that age is an independent risk factor for IS recurrence [34,35,36,37], but some did not provide confirmation [38,39]. Of note, we confirmed that the risk of long-term IS recurrence increased with age.

Diabetes mellitus is a common chronic metabolic disease and is an established risk factor for IS and coronary heart disease [40]. A meta-analysis involving 698,782 people from 102 prospective cohorts showed that diabetes is a risk factor for IS and hemorrhagic stroke [41]. In accordance with prior studies [42], we demonstrated that a significantly higher risk of IS recurrence was observed in individuals with previous IS with diabetes than in those without diabetes. Therefore, it is reasonable to think that diabetes may not only induce IS occurrence but also cause IS recurrence. Hyperlipidemia is another metabolic disorder with abnormally elevated levels of TC, TGs, and LDL, and a reduction in HDL levels. Among them, HDL is the main vasoprotective lipoprotein. Previous data have supported that serum HDL levels are inversely correlated with the incidence of IS [43]. In addition, we demonstrated that the level of HDL was negatively correlated to IS recurrence, which is in accordance with previous studies [35,43]. Numerous studies have demonstrated that LDL is a well- established risk factor for cardiovascular diseases, including IS [37,44]. Guidelines for the management of IS often emphasize the importance of controlling LDL levels via lifestyle modifications and medication interventions such as statins. The use of statins might have effectively lowered LDL levels in our study population, mitigating the impact of LDL as a standalone risk factor for stroke recurrence.

On the other hand, leukoencephalopathy, also known as white-matter hyperintensity, is a common MRI finding that shows a fluid-attenuated inversion recovery (FLAIR) sequence and an imaging manifestation of cerebral small-vessel disease [45]. To date, several research studies have consistently demonstrated that the burden of leukoencephalopathy is associated with less favorable functional outcomes in patients with IS and increased rates of symptomatic intracerebral hemorrhage [46,47,48,49]. Previously, in a retrospective analysis of 7101 patients with IS, the 1-year recurrence risk was demonstrated to be associated with leukoencephalopathy volume [50]. Moreover, Andersen et al. reported that leukoencephalopathy was significantly associated with the risk of IS recurrence [12]. Consistent with multiple previous studies, we found that leukoencephalopathy was an independent risk factor for the long-term recurrence of non-cardioembolic IS, but the prediction capabilities were not high in our cohort.

Recently, an increasing number of studies have confirmed that inflammatory responses after IS occur and persist throughout the entire brain. Brain inflammation might continuously shape evolving pathologic characteristics after IS and affect the long-term neurological prognosis for IS patients [51]. Of note, we eventually identified inflammation-associated parameters, including NE, MO, PLR, and PLT, which played primary roles in the occurrence of vascular events and the worsening of outcomes. Neutrophils, as the main type of leukocyte and major determinant of inflammation, have become a predictor of adverse outcomes in patients with vascular diseases. IS recruits neutrophils to cerebral lesions, where they damage the integrity of the blood–brain barrier and aggravate the disease process [52]. Moreover, neutrophil accumulation can significantly affect cerebrospinal fluid drainage and thus edema formation and reperfusion injury post-IS [53]. The paradoxical role of monocytes can influence stroke outcomes at different time points in the development of IS. After the onset of IS, the infiltration of CCR2+ monocytes greatly increased in mice with functional CX3CR1-CCR2 signaling, resulting in the aggravation of the acute injury. Sun et al. demonstrated that stroke-induced monocyte hexokinase 2 upregulation induced inflammatory monocyte activation, systemic inflammation, and atheroprogression via Il-1β [54]. However, in chronic IS, CCR2+ monocytes could reduce the acute brain infarct volume and brain swelling and improve functional recovery [55]. From a clinical perspective, we observed that elevated circulating monocytes could significantly decrease the long-term risk of IS recurrence. Consistently, in terms of the mechanism, the findings from other studies using RNA-seq indicated that monocytes could transform into the restorative M2 subtype over time [56,57]. The NLR, LMR, and SII are novel inflammatory biomarkers that reflect the inflammatory status in acute IS [58]. Several studies have confirmed that a higher NLR, lower LMR, and higher SII are significantly associated with poor functional outcomes and the short-term recurrence of IS [59,60,61]. However, the association with the long-term recurrence of non-cardioembolic IS remains undefined. In our study, we found that the NLR, LMR, and SII had limited prediction efficacies for the long-term recurrence for non-cardioembolic IS. This may be due to the fact that the NLR, LMR, and SII reflect the short-term inflammatory states after IS. Platelet aggregation and thrombosis exert a pivotal role in the pathogenesis of numerous diseases such as atherosclerosis and IS [62]. The platelet has been well-confirmed to be a risk factor for stroke occurrence, but a definitive result for the prognosis of stroke has not been documented. We found that the recurrence rate increased at lower platelet counts in patients with a <450 × 10^9^/L platelet count. Consistently, Yang et al. reported a multicentric prospective study that confirmed that lower (100~155 × 10^9^/L) platelet levels were significantly correlated with an increased risk of poor functional outcomes with respect to IS compared with the intermediate reference level (186~212 × 10^9^/L) [63]. Our findings may be explained in two aspects. Firstly, we found a negative correlation between platelet count and age (r = −0.1218, *p* = 0.0010, Supplement Figure S1A). Older individuals experienced a heavier atherosclerosis burden for cerebrovascular disease. Secondly, we identified a significant positive correlation with respect to WBCs (r = 0.3234, *p* < 0.0001, Supplement Figure S1B). In atherosclerosis and IS, this implies that thrombosis formation required more platelet activation and increased circulating platelet–leukocyte aggregation [64,65,66]. Notably, the PLR is a beneficial, repeatable biomarker for systemic inflammation and is related to the pathophysiological mechanism of inflammatory and hemostatic/thrombotic pathways underlying IS pathogenesis [67]. In Li et al.’s [68] study, in 185 patients exhibiting embolic strokes of an undetermined source, PLR was demonstrated to be significantly correlated with IS recurrence after a median follow-up of 2.1 years.

Some previous studies have confirmed and demonstrated that albumin levels, which have been identified to play a neuroprotective role, can decrease the risk for poor prognosis in acute IS patients [69]. However, few studies have reported the relationship between albumin levels and the recurrence of IS. Similarly, Zhang et al. [70]. illustrated that albumin levels in recurrent IS patients were significantly lower than those in non-recurrent patients, and a lower albumin level increased the risk of recurrence in acute IS patients, which is consistent with the results of our study. Bilirubin, a metabolic end-product of heme degradation, has long been regarded as a potentially toxic substance, and its elevated levels can induce irreversible damage to the brain and nervous system [71]. However, evidence strongly suggests that bilirubin has anti-inflammatory, potent antioxidant, and neuroprotective properties [72]. Of note, the prognostic values of bilirubin in stroke are controversial, since bilirubin exhibits both neuroprotective and neurotoxic effects. At least two studies validated that TBIL and DBIL levels were significantly and positively associated with stroke severity [71,73]. Moreover, Ouyang et al. [74] demonstrated that elevated levels of serum TBIL, DBIL and IBIL were significantly associated with poor functional outcomes in patients with AIS or TIA at 3-month and 1-year follow-ups. We identified that IBIL was significantly positively correlated with non-cardioembolic IS recurrence. We speculated that the possible reason for this result was that long-term chronic indirect hyperbilirubinemia may induce cerebrovascular dysfunction and damage the nervous system.

Despite these merits, our study still has several limitations and shortcomings. Firstly, the present study was a single-center, retrospective study with potential selection biases. Secondly, although the patients were randomized into training and testing sets, a large, multicenter study will need to be conducted and incorporated in the future. Thirdly, although we enrolled patients based on inclusion/exclusion criteria, avoiding information bias, selection bias, loss to follow-up and confounding factors was difficult. Fourthly, the observation that a significant proportion of subjects in our study were not taking aspirin or statins raises valid concerns about the appropriateness of the treatment. Medication adherence of aspirin or statins for secondary prevention tends to decrease over time. Factors contributing to poor medication adherence include family economic conditions, educational attainment, contraindications, and intolerance to medications. On the other hand, the results could also be due to inadequate follow-up data and insufficient sample sizes. These are the possible reasons that explain why antiplatelet drugs and statins did not reduce the recurrence risk for non-cardioembolic IS in our study. Fifthly, our study focused primarily on Asian patients, which limits the generalizability of the findings. Lastly, the developed nomogram model will need to be further validated in additional prospective cohort studies.

## 5. Conclusions

We constructed and validated a nomogram model that could accurately and precisely predict the long-term recurrence probability of non-cardioembolic IS. Furthermore, the nomogram could provide new measures for the prevention of recurrence and the clinical management of non-cardioembolic IS patients. Moreover, we also demonstrated that NE, MO, PLT, TP, ALB, IBIL and HDL were independent risk factors for non-cardioembolic IS recurrence. Lastly, given the secondary prevention of non-cardioembolic IS, identifying and actively preventing risk factors are essential for high-risk patients. Additional multi-center studies could be conducive to the application of the nomogram model for further research.

## Figures and Tables

**Table 1:** Clinical and demographic characteristics of patients in the training and testing sets.

Characteristic	All (*n* = 729)	Training (*n* = 511)	Testing (*n* = 218)	*p*-Value
Gender				0.543
Female	231 (31.7%)	158 (30.9%)	73 (33.5%)	
Male	498 (68.3%)	353 (69.1%)	145 (66.5%)	
Age(year)				0.226
<65	373 (51.2%)	269 (52.6%)	104 (47.7%)	
≥65	356 (48.8%)	242 (47.4%)	114 (52.3%)	
Smoking				0.286
No	566 (77.6%)	391 (76.5%)	175 (80.3%)	
Yes	163 (22.4%)	120 (23.5%)	43 (19.7%)	
Drinking				0.899
No	647 (88.8%)	454 (88.8%)	193 (88.5%)	
Yes	82 (11.2%)	57 (11.2%)	25 (11.5%)	
CAS				0.525
No	139 (19.1%)	103 (20.2%)	36 (16.5%)	
CIMT	103 (14.1%)	67 (13.1%)	36 (16.5%)	
Stable plaques	118 (16.2%)	79 (15.5%)	39 (17.9%)	
Unstable plaques	278 (38.1%)	199 (38.9%)	79 (36.2%)	
Carotid stenosis	91 (12.5%)	63 (12.3%)	28 (12.8%)	
Leukoencephalopathy				0.090
No	9 (1.2%)	8 (1.6%)	1 (0.5%)	
Fazekas 1 grade	458 (62.8%)	321 (62.8%)	137 (62.8%)	
Fazekas 2 grade	190 (26.1%)	125 (24.5%)	65 (29.8%)	
Fazekas 3 grade	72 (9.9%)	57 (11.2%)	15 (6.9%)	
Aspirin				0.292
No	222 (30.5%)	162 (31.7%)	60 (27.5%)	
Yes	507 (69.5%)	349 (68.3%)	158 (72.5%)	
Statins				0.213
No	211 (28.9%)	155 (30.3%)	56 (25.7%)	
Yes	518 (71.1%)	356 (69.7%)	162 (74.3%)	
Hypertension				0.031
No	166 (22.8%)	130 (25.4%)	36 (16.5%)	
Adequately controlled	463 (63.5%)	313 (61.3%)	150 (68.8%)	
Inadequately controlled	100 (13.7%)	68 (13.3%)	32 (14.7%)	
Diabetes				0.997
No	532 (73.0%)	373 (73.0%)	159 (72.9%)	
Adequately controlled	141 (19.3%)	99 (19.4%)	42 (19.3%)	
Inadequately controlled	56 (7.7%)	39 (7.6%)	17 (7.8%)	
Recurrence				0.918
No	592 (81.2%)	414 (81.0%)	178 (81.7%)	
Yes	137 (18.8%)	97 (19.0%)	40 (18.3%)	

## Data Availability

The original contributions presented in this study are included in the article material, further inquiries can be directed to the corresponding author.

## Supplementary Materials

The following supporting information can be downloaded at: https://www.mdpi.com/article/10.3390/brainsci13071051/s1, Figure S1. The correlation between PLT with age and WBC in non-cardioembolic IS patients.
