# Supplementary material for: A Nomogram for Predicting the Recurrence of Acute Non-Cardioembolic Ischemic Stroke: A Retrospective Hospital-Based Cohort Analysis
Source: Brain Sci. 2023 Jul 10;13(7):1051. doi: 10.3390/brainsci13071051 (PMC10377670; doi:10.3390/brainsci13071051)
Supplement: Supplementary file 1 [file brainsci-13-01051-s001.zip › brainsci-2437755-supplementary.pdf]

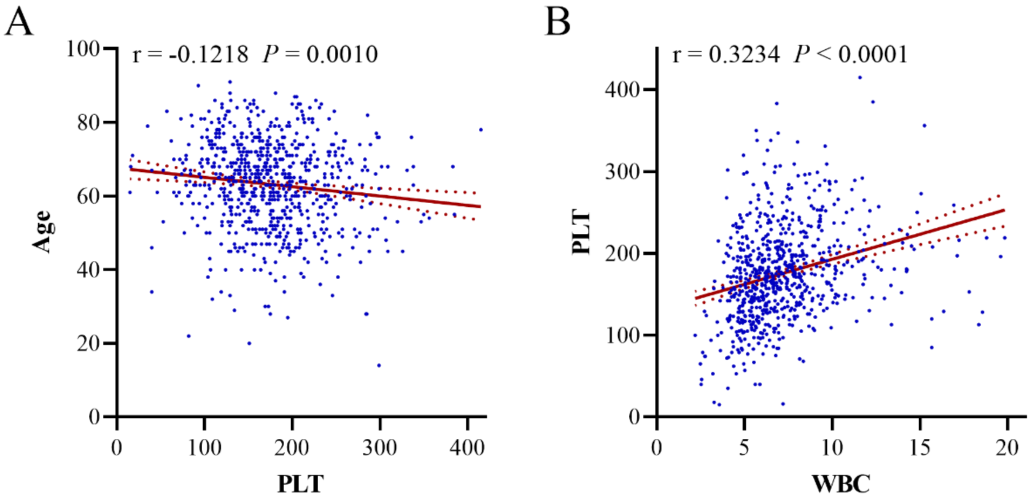

Supplement Figure S1. The correlation between PLT with age and WBC in non-cardioembolic IS patients. (**A**): PLT was negatively correlated with age (r = −0.1218, p = 0.0010). (**B**): PLT counts were significantly positively correlated with WBC counts (r = 0.3234, $p < 0.0001$).
